# Sound Insulation and Reflection Properties of Sonic Crystal Barrier Based on Micro-Perforated Cylinders

**DOI:** 10.3390/ma12172806

**Published:** 2019-08-31

**Authors:** Stefan M. Dimitrijević, Víctor M. García-Chocano, Francisco Cervera, Emelie Roth, José Sánchez-Dehesa

**Affiliations:** 1 Structor Akustik AB, Solnavägen 4, 113 65 Stockholm, Sweden; 2 School of Electrical Engineering, University of Belgrade, Bulevar K. Aleksandra 73, 11000 Belgrade, Serbia; 3 Department of Electronic Engineering, Universitat Politècnica de València, Camino de Vera s/n, ES-46022 Valencia, Spain

**Keywords:** sonic crystals, noise barriers, sound insulation, sound reflection, EN 1793-5, EN 1793-6

## Abstract

A sonic crystal barrier, consisting of empty micro-perforated cylindrical shells, was built on the campus at the Universitat Politècnica de València in 2011 and characterised by using a non-standardised measurement technique. In this paper, the sonic crystal barrier, upgraded with rubber crumb inside the micro-perforated cylindrical shells, was characterised by using standardised measurement techniques according to EN 1793-5 and EN 1793-6. As a result of the characterisation, sound insulation properties of the barrier were shown to be a combination of the absorptive properties of the individual building units and the reflective properties of their periodic distribution. In addition, its performance was compared with a similar barrier consisting of rigid polyvinyl chloride (PVC) cylinders, which was recently characterised using the same standardised techniques. In comparison with the barrier based on PVC cylinders, the barrier investigated here produced a broadband enhancement of the sound insulation and lower reflection indices in the targeted frequency range. It was also shown that the influence of leakage under the barrier and the width of the temporal window on sound insulation was negligible. While EN 1793-5 and 1793-6 allow a direct comparison of the performance of different noise barriers, the applicability to this new type of barriers requires further investigation.

## 1. Introduction

Structures consisting of sound scatterers periodically arranged in a lattice, known as sonic crystals (SCs), have been the subject of many research studies in the last two decades. Their ability to prevent sound propagation in certain frequency bands, called band gaps, is their most important characteristic. This is possible due to a mechanism called Bragg scattering, which is the destructive interference between the sound reflected from different scatterers in the lattice. A review paper by Kuswaha [1] reported the “state of the art” regarding the different theoretical approaches employed to predict acoustic band gaps in SCs updated up to 1995. Experimental characterisation of acoustic band gaps was first reported in 1995 within a kinematic sculpture made by Eusebio Sempere, which is exhibited at the Juan March Foundation in Madrid [2]. A few years later, the results were confirmed in a laboratory environment [3,4], where the existence of complete and partial band gaps was associated with the lattice symmetry and its filling fraction. Afterwards, wider and frequency-tuneable band gaps were shown to be feasible by changing the spatial arrangement and the mechanical properties of scatterers [5,6]. It should be noted that references already cited and the ones to come are strictly focused on the application of SCs in developing a new type of acoustic barriers and, therefore, the reader is addressed to recently published literature where other applications of photonic crystal and acoustic metamaterial are extensively reported [7,8].

Compared to traditional noise barriers, SCs do not necessarily need foundations since they show almost no resistance to air flow. However, a word of caution regarding this property is warranted. When the velocity of air is above a certain cut-off, the generated flow noise can quench the band gaps [9]. SCs are also lighter compared to traditional noise barriers and have great aesthetic. Nevertheless, there are very few practical implementations of SCs outside laboratory environments [10,11], mostly because of fabrication cost. However, the costs could be substantially reduced through mass production.

Experimental data show that a periodic arrangement of metallic cylinders in air provides sound attenuation of up to 25 dB [3,10,11,12,13,14], which could compete with traditional noise barriers. At band gap frequencies, simultaneous low transmittance and high reflectance are expected due to Bragg scattering. Unfortunately, the band gaps produced by SCs made of rigid cylinders are confined to a relatively narrow band of frequencies. Outside the band gaps, attenuation is very poor, making the use of SCs infeasible against broadband noise. Hence, additional physical mechanisms should be involved in order to achieve broadband attenuation. In that sense, the use of absorbing material covering the scatterers and local resonant phenomena have been recently proposed [14,15,16,17,18,19]. In this way, it is possible to obtain barriers whose frequency-dependent attenuation is more uniform and, in some cases, to enhance their overall performance.

Most of the reported research on the characterisation of SCs, both in laboratory environments [4,5,12,13,14,15,16,17,18,19] and in situ [2,10], has employed non-standardised measurement techniques, where neither diffraction nor unwanted reflections were excluded. This means that the measured attenuation values should be considered as indicative rather than precise. In this regard, the standardised EN 1793-5 [20] and EN 1793-6 [21] methods specify test procedures for assessing the intrinsic reflection and airborne sound insulation performance for noise reducing devices. For example, by applying an appropriate windowing technique, EN 1793-6 accounts for transmitted components only, while dismissing diffracted and sound reflected components from the ground and other objects located nearby. Similarly, the windowing technique in the case of EN 1793-5 excludes components other than those reflected from the barrier itself. Both methods were initially developed and tested by the European Commission funded projects ‘‘Adrienne” [22] and “QUIESST” [23]. However, the methods have been tested with traditional noise barriers only, while their applicability in the case of SCs is still uncertain.

Recently, Morandi at al. [24] characterised an SC noise barrier made of polyvinyl chloride (PVC) rods with a lattice constant of 0.2 m, by implementing standardised EN 1793-5 and EN 1793-6 methods. They showed that the values of the sound insulation index (*SI*) and the reflection index (*RI*) strongly depend on the measurement configuration, attaining maximum values of about 24 dB for *SI* and around 1 (on the decimal scale) for *RI* in the Bragg band gap. While the EN 1793-6 method allows calculation of the intrinsic airborne sound insulation, the method itself restricted the applicability to SCs with no more than three rows of cylinders in the lattice. This is due to inability of the temporal window to include all reflections with a significant spectral content. For sound reflection measurements, correlation between the number of rows and the measured data was found to be poor, according to Figure 7 in [24].

Transmittance, reflectance, and absorption properties of a SC barrier based on three rows of empty micro-perforated (MP) cylindrical shells were previously characterised by using a non-standardised measurement technique [11]. In the present research, characterisation of the SC barrier consisting of MP cylindrical shells but with an inner core made of rubber crumb, is presented. The purpose of adding the inner core was improving the absorption at high frequencies, where the rubber crumb provides better dissipation of the acoustic energy [15]. On the one hand, a modelling technique similar to that presented in [11] was employed in order to compare this barrier with the barrier based on empty MP cylindrical shells used in the previous characterisation. On the other hand, the standardised EN 1793-5 and EN 1793-6 measurement methods were implemented in order to compare data with the barrier based on rigid PVC cylinders, which was characterised in the same manner [24].

The paper is described as follows. First, Section 2.1 reports analysis of the physical mechanisms involved in the attenuation properties of the SC barrier with MP cylindrical shells in comparison with the barrier solely based on empty MP shells. Then, Section 2.2 presents a brief description of the standardised EN 1793-5 and EN 1793-6 measurement methods employed here. Later, in Section 2.3, theoretical analysis in the framework of finite element method using both plane- and cylindrical-based approaches is reported. A comprehensive report on the experimental data measured by using the standardised measurement methods on the SC barrier with MP cylinders and rubber crumb is presented in Section 3. To better quantify the effects of micro-perforations on the barrier performance, measured data are compared with those resulting from the standardised characterisation of an SC barrier made of solid PVC cylinders [24]. Concluding remarks are presented in Section 4, where superior performance of the barrier based on MP cylinders is understood in terms of the absorptive properties of its building units. The resilience of the constructed MP barrier to deterioration due to ageing is also highlighted. The micro-pores remained open eight years after barrier installation in open air. Finally, the appendices present details of the model employed to describe the absorbing units (Appendix A), the influence of the width of the temporal window on the sound insulation values (Appendix B), and how the leakage under the barrier affects the sound insulation (Appendix C).

## 2. Materials and Methods

### 2.1. SC Noise Barrier Based on Micro-perforated Cylindrical Shells and Rubber Crumb Inside

**Figure 1:**
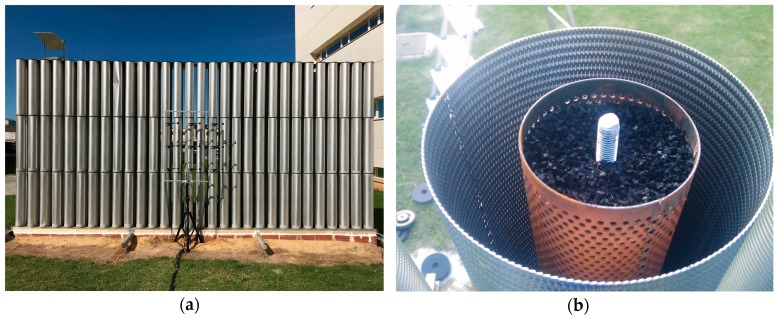
Sonic crystal (SC) barrier sample implemented on the campus of Universitat Politècnica de València: (**a**) Appearance of SC barrier sample made of three rows of absorbing cylindrical units, along with experimental setup; (**b**) internal cross-section of a cylindrical unit, consisting of a porous core made of rubber crumb and outer micro-perforated aluminium shell.

A SC noise barrier consisting of a square distribution of empty MP cylindrical shells was constructed and characterised by researchers of Universitat Politècnica de València (UPV) in 2011 [11]. The building units of the barrier investigated in this study are slightly different to those employed in [11], but their space arrangement remained the same. Namely, the barrier, which is shown in Figure 1a, is made of a total of three rows, each having 30 cylindrical units with a height of 3 m and an external radius of 8 cm. The lattice period was 0.22 m and the corresponding filling fraction of the scatterers was 41.5%. The actual size of the barrier sample was 6.54 × 0.6 × 3 m. The cylinders were placed on a metal base, which was located at about 0.17 m from the ground. Given the value of the lattice period, the Bragg band gap was expected to be centred at approximately 780 Hz, where the lattice period equals one-half wavelength.

Figure 1b shows the internal cross-section of the upgraded cylindrical building units comprised of rubber crumb as the porous core and an MP aluminium shell as the outer layer. The porous core consisted of rubber crumb inserted into a perforated steel cylinder with a radius of 4 cm and a thickness of 1 mm. The diameter of the perforations on the steel cylinder was 3 mm and the cylinder perforation ratio was 32.6%, making the cylinder acoustically transparent [15]. Thus, it allowed sound to interact with the rubber crumb. The purpose of the inner core is to dissipate high-frequency components of the acoustic energy penetrating the micro-perforations of the external aluminium shell.

The outer MP layer was made of an aluminium plate with a thickness of 1 mm and a perforation ratio of 3.3%. Since the micro-perforations were punched, the resulting pore geometry was complex. However, according to research with similar plates [25,26], the perforations of the MP layer could be modelled as rectangular apertures with an effective length of 1.11 mm and effective width of 95 µm. Characterisation of this type of MP plates in an impedance tube revealed broadband reflection and absorption spectra [11].

In what follows, attenuation properties of four different barrier samples are shown, which were constructed and analysed by using the methods already explained in [11]. Sample 1 was named according to that previously studied in [11] and consisted of three layers of empty MP cylindrical shells. The barrier sample 2 corresponds to a barrier made of one layer of MP cylinder with a porous core made of rubber crumb, as explained above. This type of cylindrical scatterer was also employed to construct sample 3 and sample 4, consisted of two and three layers of scatterers, respectively.

**Figure 2:**
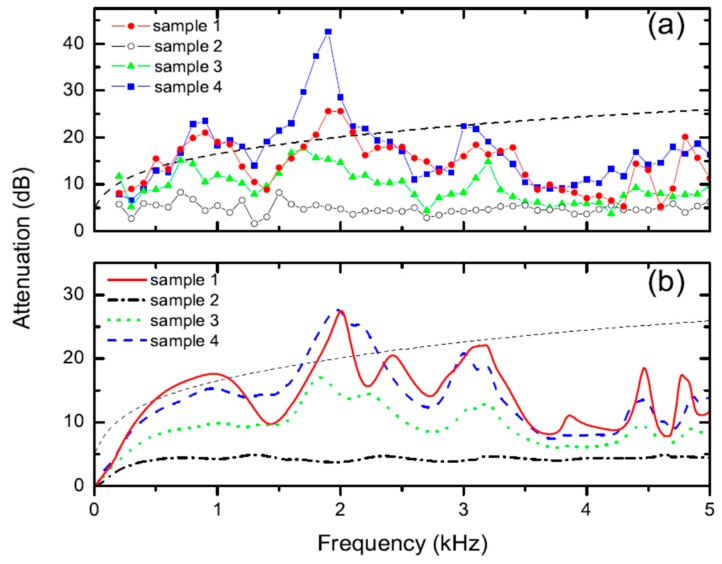
(**a**) Attenuation spectra measured for several SC barrier samples implemented on the campus at the Universitat Politècnica de València; (**b**) corresponding calculated spectra simulated according to procedure described in [9].

Figure 2 shows a comparison of the attenuation obtained for the four samples previously described. Figure 2a shows the experimental data while Figure 2b shows the numerical simulation using the multiple scattering method. Attenuation was calculated in dB as −10log (T), where T is the measured or simulated transmittance. The reader is addressed to [11] and references therein in order to obtain detailed description of the measurement procedure as well as the simulation method employed to obtain the transmittance. On the one hand, it is observed in Figure 2 that attenuation of the barrier increased with the number of layers. On the other hand, the attenuation for Sample 1 and Sample 4, both having the same thickness, is similar except that barrier containing rubber crumb (Sample 4) apparently showed a slightly larger attenuation. This is mainly visible at the peak centred at 2 kHz, where the attenuation was broader and higher. This conclusion is not fully described by the numerical simulations, which only explain the peak broadening. The grey dashed line corresponds to the calculated performance of a rigid wall having the same external dimension as the SC barriers investigated here.

It is interesting to point out that the measured attenuation shown in Figure 2 overcame the one obtained for similar SC barrier based on cylinders made of porous materials [15,16], showing smaller absorptive properties at low frequencies. Also, it is remarkable that the band gap, which was expected to be located at approximately 780 Hz, appeared as a broad peak due to the high absorption produced by the micro-perforations in this region.

Hereafter, a further analysis of barrier sample 4 is reported but using standardised measurement techniques (see Section 2.2) and a modelling based on the finite element method (see Section 2.3). Thus, the measurement techniques and the numerical simulations were different to those employed in this section. The goal was to give a direct comparison of the performance of the SC barrier with MP cylinders with another based on PVC solid cylinders reported in [24].

### 2.2. Measurement Methods

European standards EN 1793-5 [20] and EN 1793-6 [21] specify test methods for in situ assessment of reflection and insulation performance, respectively, of road traffic noise-reducing devices. The methods are used to check intrinsic characteristics of reflection and airborne sound insulation of noise-reducing devices to be installed along roads and those already in place. They are also applied to examine barrier design specifications, verifying long-term barrier performance, and facilitating interactive designing of new products. Such measurements are widely accepted as a normalized way of noise-reducing device characterisation. The advantage of in situ methods over traditional laboratory measurements, EN 1793-1 [27] and EN 1793-2 [28], is that they can be undertaken in the presence of traffic background noise. This is possible by using deterministic excitation signals in combination with impulse-response measurement techniques. Also, in situ measurements assume a directional sound field compared to a diffuse field in the case of laboratory measurements, since the former is more realistic. Thus, results are comparable but not identical [29,30]. In situ methods use the same principles and equipment for both *RI* and *SI* measurements. The main principles of the methods are explained in more detail below, in connection with the actual SC barrier sample.

#### 2.2.1. EN 1793-6 in Situ Insulation Method

Two impulse-response measurements are the basis of the in situ insulation method. The first is performed in a free field without a noise barrier and the second with a noise barrier. Both measurements are carried out keeping the same loudspeaker-to-receiver distance, calculated by summing the loudspeaker-to-barrier and barrier-to-receiver distances with the thickness of the noise barrier. Both loudspeaker-to-barrier and barrier-to-receiver distances are defined by the method itself and the values are 1 m and 0.25 m, respectively. The receiver in the present case was a 3 × 3 microphone grid (M1–M9), with any two adjacent microphones spaced 0.4 m apart.

**Figure 3:**
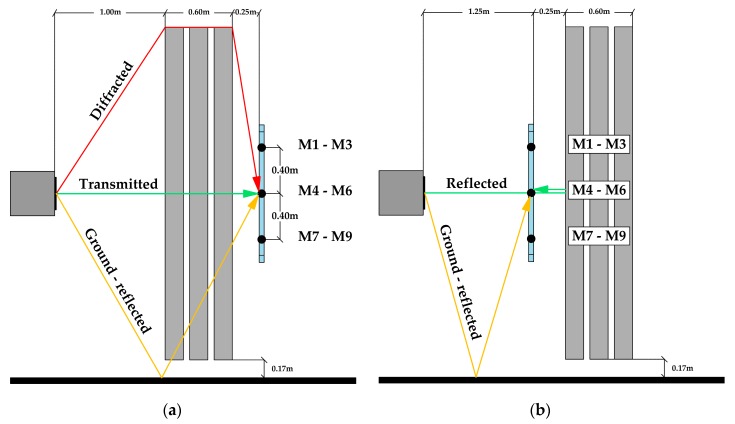
Illustration of standardised EN 1793-5 and EN 1793-6 measurement methods in the presence of the SC barrier: (**a**) Experimental setup for *SI* measurement; (**b**) experimental setup for *RI* measurement.

When performing a measurement in the presence of a noise barrier, the sound emitted by the sound source is partly reflected, partly absorbed, partly transmitted, and partly diffracted by the barrier itself. To calculate the intrinsic characteristic of the barrier, all sound components, except those directly transmitted through the barrier, are discarded. This refers to both diffracted and ground-reflected sound waves (see Figure 3a). It is achieved by applying a specially designed temporal window called the *Adrienne* temporal window. Finally, the energies of the time windowed impulse responses are calculated and compared to those from the free field measurement. The measured data are averaged over all nine microphones and a specific quantity called the sound insulation index (*SI)* is calculated:

(1) $$SI_{j}\left(\mathrm{dB}\right)=-10\log\left\{\frac{1}{n}\sum_{k=1}^{n}\frac{\int_{\Delta f_{j}}{\left|F\left[h_{t,k}\left(t\right)w_{t,k}\left(t\right)\right]\right|}^{2}\mathrm{df}}{\int_{\Delta f_{j}}{\left|F\left[h_{i,k}\left(t\right)w_{i,k}\left(t\right)\right]\right|}^{2}\mathrm{df}}\right\},$$

where $h_{i,k}\left(t\right)$ is the free-field impulse-response at the k-th microphone position, $h_{t,k}\left(t\right)$ is the impulse-response at the k-th microphone position with the barrier in between, $w_{i,k}\left(t\right)$ and $w_{t,k}\left(t\right)$ are the time windows (*Adrienne* temporal windows) for the free-field and the transmitted components, respectively, at the k-th microphone position, and F denotes the Fourier transform. Finally, index j denotes the j-th one-third octave frequency band with bandwidth $\Delta f_{j}$ and the integer n (n=9) defines the number of microphone positions.

Single-number ratings are calculated in addition to the frequency-dependent *SI* indices presented in Equation (1). The single-number ratings are defined by EN 1793-6 standard and present a standard way of rating the performance of noise barriers. They were introduced because there was a need to categorise the airborne sound insulation performance of noise barriers. In that way, formulation of acoustic requirements in noise barrier specification was simplified. In general, three single-number values are derived: One for acoustic elements, one for posts, and a global rating. A post is defined here as a pillar holding two adjacent acoustic elements together. To calculate single-number values for the acoustic elements and posts, corresponding individual single-number values are weighted according to the normalized traffic noise spectrum defined in EN 1793-3 [31]. The global rating is a logarithmic average between single-number ratings for the acoustic element and the post. Since there are no posts in the case of SCs, only one single-number rating was calculated, without any global rating.

The size of the barrier affects the width of the *Adrienne* temporal window as well as the lowest reliable frequency f_MIN_ at which the barrier can be characterised. On the first hand, this stems from the fact that the low-frequency limit of EN 1793-6 in situ insulation method is inversely proportional to the width of *Adrienne* temporal window. As an indication of the low-frequency limit, the first notch in the magnitude spectrum of the *Adrienne* window is used [29]. On the other hand, the width of the Adrienne window depends on the temporal delay between direct sound and diffracted/ground reflected sound. The size of the barrier will affect the temporal delay and, thus, the width of the Adrienne window and low-frequency limit. The larger barrier sample, the lower f_MIN_, hence the wider frequency range of *SI* is reported. The influence of the size of the barrier is well explained in [32]. To calculate the width of the *Adrienne* temporal window, the lowest latencies between the diffracted and ground-reflected sound need to be determined relative to the transmitted sound, for all individual microphone positions. To simplify the calculation of the width of the Adrienne temporal window, the present study assumed that all sound components at three individual microphones in the same row of the microphone grid arrived at the same time.

#### 2.2.2. EN 1793-5 in Situ Reflection Method

The measurement of sound reflection is also based on two impulse-response measurements, but for different configurations. Namely, both the loudspeaker and the microphone grid are placed on the same side of the sample being tested, at distances of 1.5 m and 0.25 m from the sample, respectively. The windowing technique in this case is applied in a similar manner, but unlike the *SI* measurement, only the ground-reflected sound wave is discarded (see Figure 3b). By comparing the energies of the reflected component of the impulse responses taken in front of the sample and the incident component of the free-field impulse response, this method defines a specific quantity called the reflection index-*RI*:

(2) $${RI}_{j}=\frac{1}{n_{j}}\sum_{k=1}^{n_{j}}\left[\frac{\int_{\Delta f_{j}}{\left|F\left[h_{r,k}\left(t\right)\cdot w_{r,k}\left(t\right)\right]\right|}^{2}\mathrm{df}}{\int_{\Delta f_{j}}{\left|F\left[h_{i,k}\left(t\right)\cdot w_{i,k}\left(t\right)\right]\right|}^{2}\mathrm{df}}\cdot C_{\mathrm{geo},k}\cdot C_{\mathrm{dir},k}\left(\Delta f_{j}\right)\cdot C_{\mathrm{gain},k}\left(\Delta f_{g}\right)\right],$$

where $h_{i,k}\left(t\right)$ is the incident reference component of the free-field impulse response at the k-th measurement point, $h_{r,k}\left(t\right)$ is the reflected component of the impulse response taken in front of the sample at the k-th measurement point, $w_{i,k}\left(t\right)$ and $w_{t,k}\left(t\right)$ are the time windows (*Adrienne* temporal windows) for the free-field and the reflected components, respectively, at the k-th microphone position, F denotes the Fourier transform, j is the index of the j-th one-third octave frequency band, $n_{j}$ is the number of microphone positions on which to average ($n_{j}\geq 6$), and $\Delta f_{j}$ is the width of the j-th one-third octave band frequency band. For the k-th microphone position, $C_{geo,k}$ is the correction factor for geometrical divergence and $C_{dir,k}$ is the correction factor for sound source directivity. Finally, $C_{gain,k}$ is the correction factor to account for any change in the amplification settings of the loudspeaker and the sensitivity settings of the individual microphones, when the measurement configuration is changed from free field to in front of the sample or vice versa, with frequency range $\Delta f_{g}$ expressed in one-third octave frequency bands between 500 Hz and 2 kHz.

As in the case of sound insulation measurement, a single-number rating *DL_RI_* is delivered in addition to the frequency-dependent *RI* indices presented in Equation (2) to indicate the performance of the product. The individual sound reflection indices are weighted according to the normalized traffic noise spectrum defined in EN 1793-3 [31]. The higher single-number rating, the higher reflective properties of the barrier.

The signal subtraction technique [33] is applied to separate a reflected component on each microphone of the grid. The technique disregards direct sound waves from both the free-field and the measurement with the barrier. The sound reflection index, *RI*, is a quantity different from the sound absorption coefficient obtained by the laboratory method EN 1793-1 [27]. However, these two quantities can be converted into each other, taking the complement to one. Even though the two methods imply different sound fields (directional field in the in situ and diffuse field in the laboratory method), there is a certain correlation between them as described in [29,34].

The equipment used to carry out both *SI* and *RI* measurements consisted of a *Roland Studio-Capture* multichannel sound card, *Mackie SRM150* single driver loudspeaker, and nine *NTI Audio M4261* microphones supported by a custom-made aluminium frame. The measuring platform *EASERA* (AFMG, Berlin, Germany) was used for data acquisition. Measurements were performed by using logarithmic sine sweep excitation signal with a length of 5.5 s. The *MATLAB* (The MathWorks, Inc., Natick, MA, USA) computing environment was employed for evaluation purposes.

### 2.3. Finite Element Method

Numerical simulations were performed using the *Acoustics Module* of the commercial software package *COMSOL Multiphysics* (The COMSOL Group, Stockholm, Sweden). Two-dimensional (2D) simulations based on the finite element (FE) method were conducted. On the one hand, the acoustic band structure of a square lattice of rigid cylinders was calculated in order to determine the band gaps along the principal directions in the lattice. On the other hand, the attenuation produced by the barrier was calculated by considering two types of impinging sound waves: Plane wave and cylindrical wave. The corresponding results for the case involving a barrier made of MP cylinders is also presented for comparison purposes.

#### 2.3.1. Acoustic Bands

**Figure 4:**
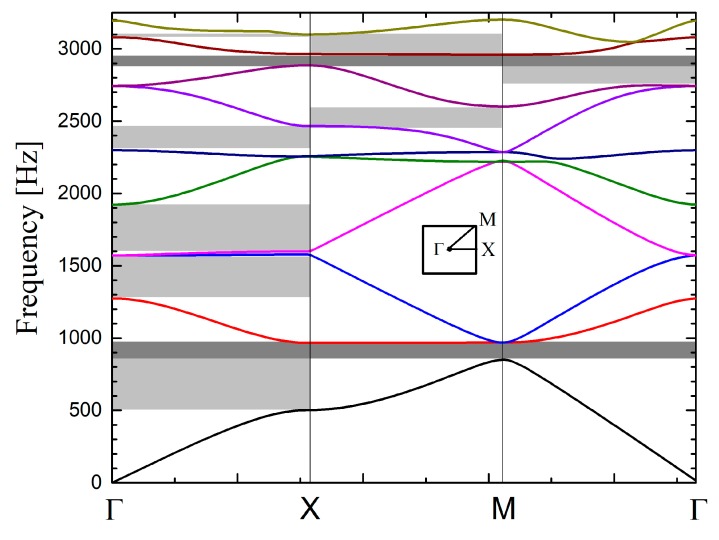
Acoustic bands of a square lattice of rigid cylinders embedded in air. Dark stripes indicate complete band gaps and light grey stripes define the partial gaps. The inset plots the reciprocal lattice together with high symmetry points that define the irreducible region.

To compute the band diagram of a square lattice of rigid cylinders, a single unit cell was modelled in *COMSOL Multiphysics*. This cell comprises a single cylinder in a squared domain of air whose dimension was defined by the lattice constant. Floquet conditions were configured on the four external boundaries of the unit cell and an eigenfrequency analysis was performed at wave vectors contained along the principal directions in the lattice. Figure 4 shows the calculated bands along with the definitions of the directions of the wave vector in the reciprocal lattice (see the inset).

The dark and light grey stripes in Figure 4 define complete and partial band gaps, respectively. The first complete band gap covers the frequency region of 850–970 Hz while the second covers the interval from 2890 Hz to 2965 Hz. In addition to the complete band gaps, four partial band gaps or pseudo gaps appear along the direction ΓX. This is the preferred direction for efficient acoustic barrier design, as implemented in [10,11,12,13,14,15,16].

#### 2.3.2. Attenuation by Barriers Made of Rigid Cylinders: Plane Waves Vs. Cylindrical Waves

Let us first consider the case of plane waves (PW) impinging on a structure composed of three rows of rigid cylinders, each row having an infinite number of cylinders. This scheme was simulated through a 2D section of the barrier in which the unit cell contained three cylinders and where the boundaries were configured with periodic conditions. The dimension of such a domain equalled the lattice constant of the structure, while the cylinders were considered infinitely long along their axis. Excitation was provided by a plane wave radiation condition at the left and right outer boundaries. This non-reflecting boundary condition allowed the plane wave to enter and leave the domain without any significant reflections. Two different physical magnitudes were calculated with this scheme:

- *Pressure*: Its value can be calculated directly from the positions where the microphones are placed in the experimental setup. In this manner, values comparable to the experiments or the simulations with cylindrical waves can be computed. Normalization to a free-field measurement is not required since the amplitude of the impinging PW is one.
- *Attenuation*: Reflectance and transmittance were calculated by integrating the acoustic intensity that crosses the left and right boundaries of the air-domain, where incident, reflected, and transmitted waves enter or leave. The advantage of this computation is that the spatial energy distribution of the high-order modes excited in the sonic crystal above the diffraction limit does not produce artefacts in the frequency responses. With respect, attenuation, the quantity of interest here, is calculated as
  (3) $$\mathrm{Attenuation}\left(\mathrm{dB}\right)=-10\times log_{10}\;\left(T\right).$$

A frequency domain analysis with a sweep from 60 Hz to 5700 Hz was undertaken in both cases. The results were post-processed with *MATLAB* scripts to calculate related parameters, such as parameters integrated in one-third octave bands.

Regarding the case of impinging cylindrical waves (CW), a structure with the same number of cylinders as the actual barrier (see Figure 1a) was embedded into an air domain with dimensions of 3 × 7 m. Excitation was provided through a point source located at the same position as the loudspeaker in the experimental setup. The outer boundaries of the air domain are configured with a cylindrical wave radiation condition, which allowed waves to leave the domain without any significant reflections. The pressure was calculated at those points where the microphones were positioned in the experiments. These values were normalized to the pressure calculated under the same conditions with no barrier.

**Figure 5:**
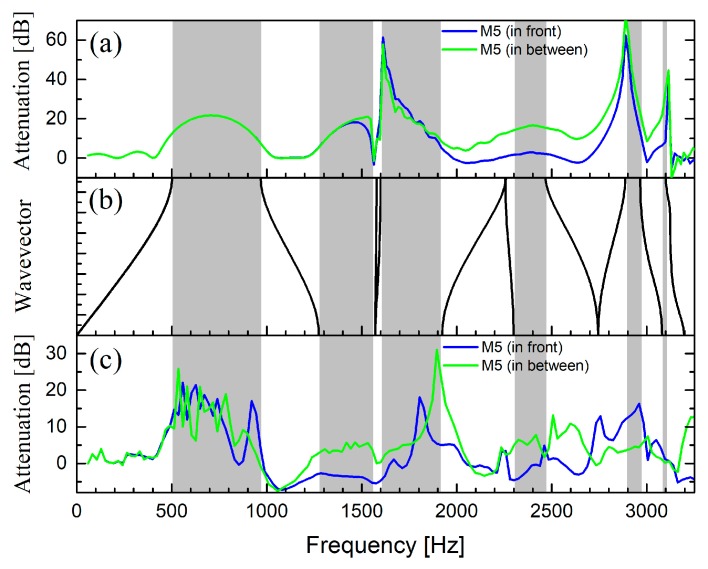
Attenuation spectra calculated for an acoustic barrier consisting of three rows of rigid cylinders arranged in a square configuration with a lattice constant of 22 cm and a volume filling ratio of 41%. (**a**) Results for incident plane waves; (**b**) band dispersion relation along the ΓX direction. Grey zones define band gaps along this particular direction; (**c**) results for impinging cylindrical waves.

Figure 5a,c shows the attenuations spectra calculated using impinging PW and CW, respectively. The spectra were calculated at two different positions, corresponding to the two possible positions of microphone M5 in: “in front” and “in between” the cylindrical scatterers, which were considered infinite along their axis. For comparison purpose, Figure 5b shows the dispersion relation along the direction X, perpendicular to the barrier surface.

It is apparent in Figure 5a that the attenuations are larger for frequency regions where there are band gaps in the dispersion relation (see Figure 5b), although the barrier contains only three scattering units along this direction. A Fano-like profile was observed at around 1600 Hz, coincident with two flat bands in the dispersion relation, which is an interference phenomenon due to the presence of a resonant state in the three-layer-thick barrier. Note that slight differences do exist between the profiles of the two observation points. The differences started appearing after the diffraction limit of the lattice, which was around 1275 Hz.

With respect to the spectra calculated with impinging CW (Figure 5c), the large attenuation regions roughly corresponded to frequencies where the band dispersion relation showed band gaps (grey zones in Figure 5b), as for the case of incident PW. Figure 5c shows negative values, which correspond to sound enhancement. Note that the case of PW also exhibited negative values around the same positions, although they cannot be properly visible due to the scale of Figure 5a. This behaviour was revealed because of the interference effect between high-order diffracted modes that propagate above the diffraction limit of the SC.

**Figure 6:**
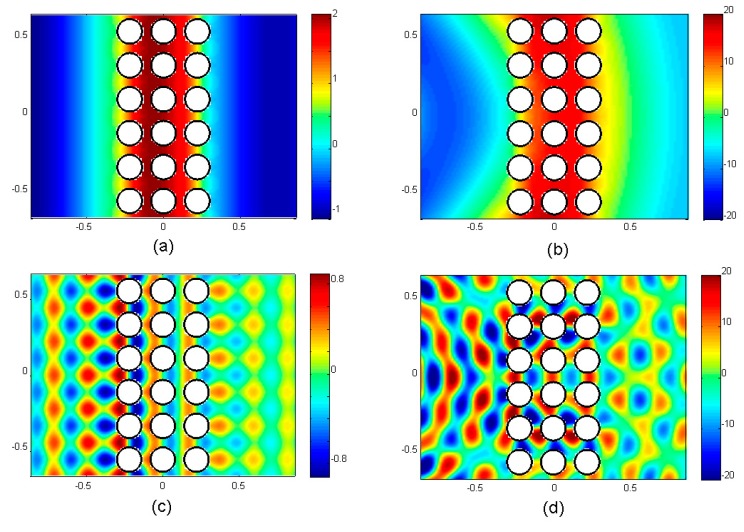
Snapshot of total pressure maps calculated when a plane (left panels) and a cylindrical (right panels) wave interacts with acoustic barrier consisting of three rows of rigid cylinders ordered in a square configuration with lattice constant of 22 cm and volume filling ration of 41%. (**a**,**b**) Calculated maps for waves with frequency of 200 Hz; (**c**,**d**) corresponding maps for waves with frequency of 1.55 kHz. Maps are calculated by using the finite element method.

The case with no interference between high-order diffracted modes is illustrated in Figure 6a,b, corresponding to an impinging PW and a CW, respectively. The pressure maps were calculated for sound waves with frequency of 200 Hz interacting with a barrier made of rigid cylinders. The abscissa and the ordinate in Figure 6 represent distances in meters, while the colour scale represents the relative pressure value. Both cases showed that, after passing through the barrier, the wave had an almost plane wavefront and therefore any measurement of the transmitted pressure levels did not depend on the position of the microphone on the vertical axis of the figure. The case with interference is illustrated in Figure 6c,d, representing impinging waves with frequency of 1.55 kHz and having plane and cylindrical wavefronts, respectively. Since the frequency is above the diffraction limit, the excited modes with components along the surface produce an interference pattern with regions where pressure increases or decreases. Now, the position of the measurement point resulted in different pressures depending on the vertical axis of the figure. In addition, samples simulated with CW were finite, and diffraction around their borders might have enhanced the interference phenomena [3,4].

#### 2.3.3. Attenuation by Barriers Made of Absorptive Cylinders: Plane Waves Vs. Cylindrical Waves

**Figure 7:**
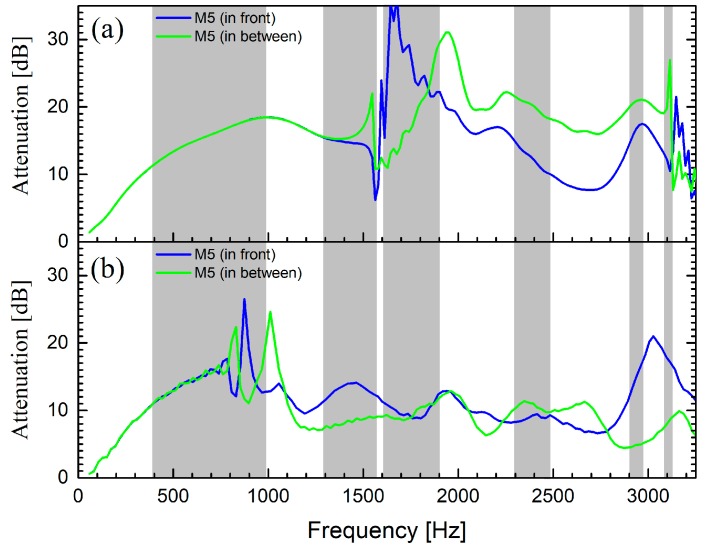
Attenuation spectra calculated for an acoustic barrier consisting of three rows of absorptive cylinders arranged in a square configuration with a lattice constant of 22 cm and a volume filling ratio of 41%. (**a**) Results for incident plane waves; (**b**) results for impinging cylindrical waves. Grey zones define band gaps along ΓX orientation (see Figure 4) of the barrier.

For a barrier based on absorptive cylindrical scatterers, Figure 7a,b shows the results of numerical simulations using incident PW and CW, respectively. The reader is addressed to Appendix A for a short portrayal of the model employed to describe the absorptive units in the numerical simulations. As in the case of barriers based on rigid cylinders, the PW calculations considered an infinite barrier in the lateral dimension, while the CW calculations consider a finite barrier with a lateral dimension equal to the constructed barrier. For discussion purposes, the grey zones define the band gaps calculated for a square lattice of rigid cylinders (see Figure 5b).

For the case of an impinging PW, it is apparent in Figure 7a how the attenuation is broadband, covering the frequency regions where sound propagation is allowed for rigid structures (white zones). Then, in comparison with the barrier based on rigid cylinders, the barrier based on MP cylindrical shells enhanced the attenuation in the broadband region due to the energy dissipation at the micro-perforations on the shell as well as at the absorptive inner core. Differences between the attenuation spectra measured at the two microphone positions, “in front” and “in between”, respectively, appeared after the diffraction limit. The diffraction limit is located around the frequency where the wavelength equals the lattice parameter, which is 1559 Hz in this case. Regarding the case of impinging CW, it was observed in Figure 7b that responses “in front” and “in between” started to differ at lower frequencies, which indicates that diffracted modes appeared earlier when cylindrical waves were employed. Note that in both cases, the attenuation spectra do not show negative values, as it was shown for the barrier based on rigid cylinders (see Figure 5a,c). Here, the absorption effects in the cylindrical scatterers significantly reduced the amplitude of the transmitted sound in such a manner that even when considering the interference of diffracted modes, the overall attenuation remained positive. In summary, a comparison of the attenuation spectra shown in Figure 5 and Figure 7 supports the construction of acoustic barriers based on absorptive cylinders like the one proposed here, rather than those based solely on rigid cylinders.

## 3. Results and Discussion

**Figure 8:**
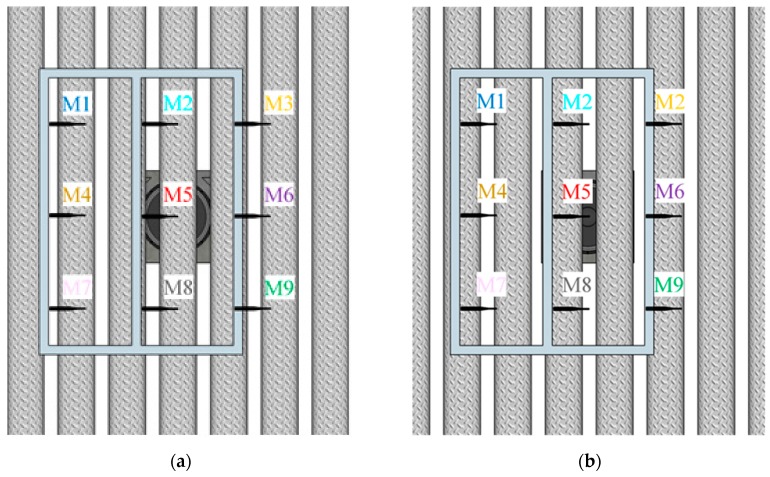
Positions of the microphone grid in two different configurations: (**a**) Configuration A, the middle column of microphones faces the centre of the cylinders; (**b**) configuration B, the middle column of microphone grid faces the centre of the space between two adjacent cylinders.

For the given size of the SC barrier sample 4 described in Section 2.1, the calculated low-frequency limit was f_MIN_ = 259 Hz. Hence, the results of the standardised characterisation are presented in one-third octave bands, starting from a central frequency of 315 Hz. The values in the lower one-third octave bands are retained for presentation purposes, as recommended by EN 1793-5 [20] and EN 1793-6 [21] standards. The results for both reflection and airborne sound insulation measurements are reported for the two configurations represented in Figure 8. In configuration A, the middle column of microphone grid faced the centre of a cylinder, whereas in configuration B, it faced a space between two adjacent cylinders. Bearing in mind the distance of 0.4 m between micro-phones in the microphone grid and the lattice parameter of 0.22 m, the spacing between the microphones was not a multiple of the lattice constant.

### 3.1. Experimental Results of Sound Insulation Index SI

**Figure 9:**
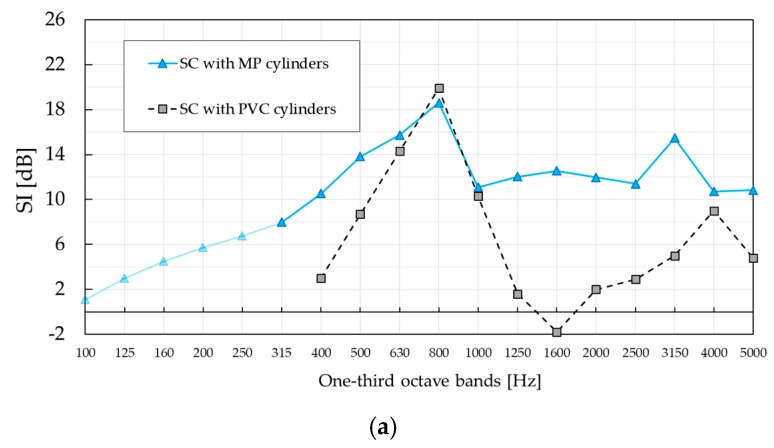

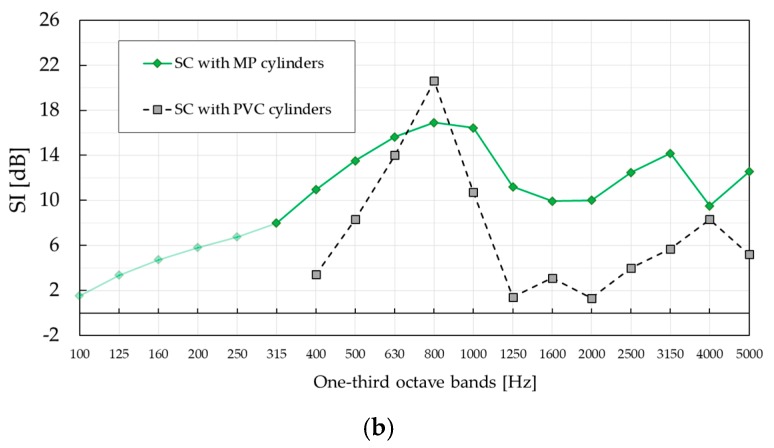
Sound insulation index sound insulation index (*SI)* of SC barrier with micro-perforated (MP) cylinders measured in two different configurations. Results are compared with those of standardised characterisation of SC barrier with PVC cylinders presented in Figure 6 in [24]. (**a**) Sound insulation index *SI*, configuration A; (**b**) sound insulation index *SI*, configuration B.

Figure 9 shows the results of the standardised characterisation of the SC barrier with MP cylinders for both configurations, A and B, calculated according to Equation (1). For the sake of comparison, Figure 9 also shows the results corresponding to the standardised characterisation of the SC barrier with PVC cylinders provided in [24], calculated in the same manner. Even though the lattice constants are slightly different, 0.22 m in the case of SC with MP and 0.2 m in the case of SC with PVC cylinders, Bragg gaps are expected to be within the same one-third octave band with a centre frequency of 800 Hz.

The *SI* peak values in both configurations correspond to a complete band gap, as predicted from the lattice constant. Those values were determined to be 18.6 dB and 16.9 dB for configuration A and B, respectively, of SC with MP cylinders. Also, the *SI* values in the complete band gap are similar to those of SC with PVC cylinders, just slightly lower. This is believed to be due to the absorptive nature of the building units, which slightly dampens the effect of the Bragg scattering. However, *SI* curves around the complete band gap are wider in case of SC with MP cylinders because of the absorption mechanism introduced by the MP layer, showing a significant difference at low frequencies. Additionally, the *SI* peak in configuration B is ‘smeared out’ towards the 1000 Hz region, making the difference in the complete band gap between the two SC barriers even more obvious.

**Figure 10:**
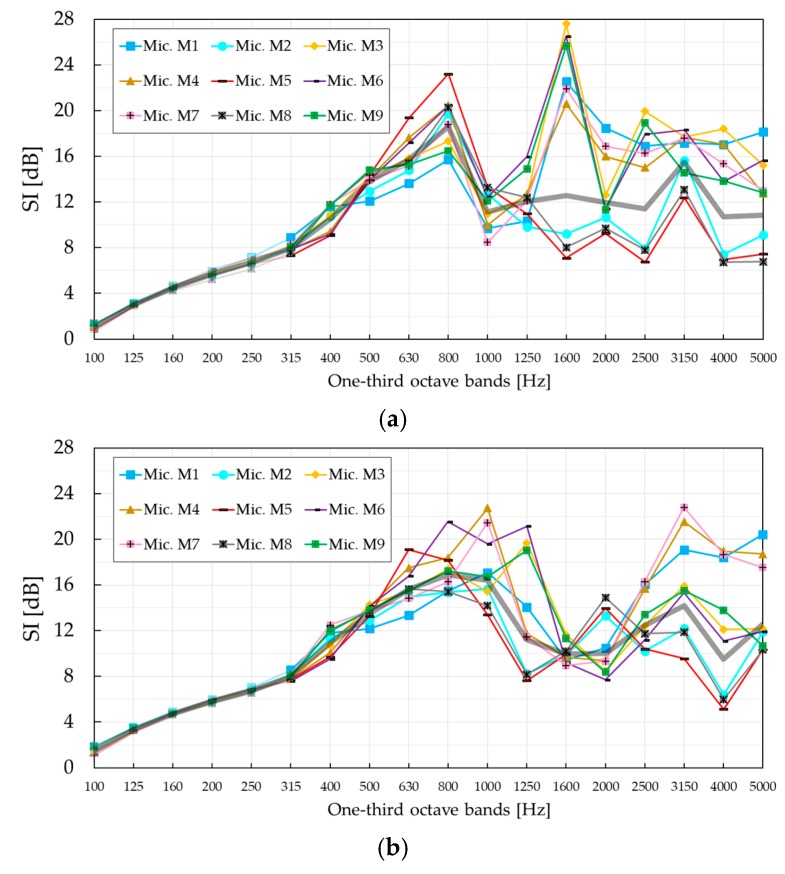
*SI* values measured at individual microphone positions M1–M9. Thick black curve shows the averaged *SI* values over all nine microphones. (**a**) Sound insulation index *SI*, configuration A; (**b**) sound insulation index *SI*, configuration B.

The presence of the absorbing MP layer was even more noticeable in the frequency region 1250–5000 Hz, showing a maximum difference of 14.4 dB in the one-third octave band with a central frequency of 1600 Hz, in the case of configuration A. Low *SI* values at twice the Bragg frequency measured for the SC barrier with PVC cylinders, negative in configuration A and around zero in configuration B, significantly differ from those measured for the SC barrier with MP cylinders. Around this frequency, the first diffracted mode become propagative, according to the band diagram in Figure 4. However, this was barely visible only in configuration A. The reason is that the *SI* curve is the result of averaging over nine microphones. This is most evident when *SI* values are investigated at individual microphone positions, as shown in Figure 10. Despite the fact that high *SI* values were measured at as many as six microphones at 1600 Hz in configuration A, the contribution of low *SI* values at the same frequency to the overall *SI* values was disregarded after averaging. A similar effect is noticeable at 3150 Hz in configuration B, where a new diffracted mode starts propagating but this time it was more apparent in the averaged *SI* curve.

The maximum *SI* value of 23.2 dB at the Bragg frequency in configuration A was measured for microphone position M5. In configuration B, the maximum *SI* value of 21.5 dB at the Bragg frequency was measured for microphone position M6. However, these values were not the maximum values for all microphone positions and frequencies. The maximum *SI* value of 27.6 dB in configuration A was measured for microphone position M3 at 1600 Hz. In configuration B, the maximum *SI* value of 22.8 dB was measured for microphone position M7 at 3150 Hz, which was also near the cut-off frequency of the diffracted mode. The diverse *SI* curves between individual microphone positions in both configurations were a consequence of the occurrence of different modes above the diffraction limit. Asymmetry of absorption properties of different cylinders, but also inherently imperfect positioning of the microphone grid, might have been additional reasons. Despite the differences in the *SI* values between different microphone positions, characteristic frequency phenomena like the Bragg gap and diffracted modes were still easily noticeable. The method itself will average out all large deviations between the *SI* values in the same frequency range, providing overall information about the effectiveness of the SC barrier. It is still very important to be aware of the strong position-dependent insulation behaviour of SCs.

As explained in Section 2.2.1, a single-number rating, *DL_SI_*, is introduced by EN 1793-6 standard in order to categorise the airborne sound insulation performance of noise barriers. Single-number rating values were calculated for different low frequency limits, f_MIN_ = 315 Hz in the case of SC with MP and f_MIN_ = 400 Hz in the case of SC with PVC cylinders. Low-frequency limits influenced calculated single-number values, since they were obtained for different frequency ranges. The calculated single-number values of SC with MP cylinders are *DL_SI, Conf-A_* = 12.3 dB and *DL_SI, Conf-B_* = 12.2 dB, while the values of SC with PVC cylinders were *DL_SI, Conf-A_* = 3.7 dB and *DL_SI, Conf-B_* = 5 dB.

Requirements for sound attenuation and frequency span of the SC barrier needed in practice are a matter of type of noise as well as sound levels for which the barrier is used for. According to categorisation of airborne sound insulation presented in EN 1793-6, there are five possible categories (see Table 1). For given single-number values of SC with MP cylinders, *D_LSI, Conf-A_* = 12.3 dB and *D_LSI, Conf-B_* = 12.2 dB, the SC barrier is categorised as D1 against traffic noise. To rate the SC barrier against other types of noise, appropriate spectrum should be used for calculation of single-number values.

### 3.2. Experimental Results of Sound Reflection Index RI

**Figure 11:**
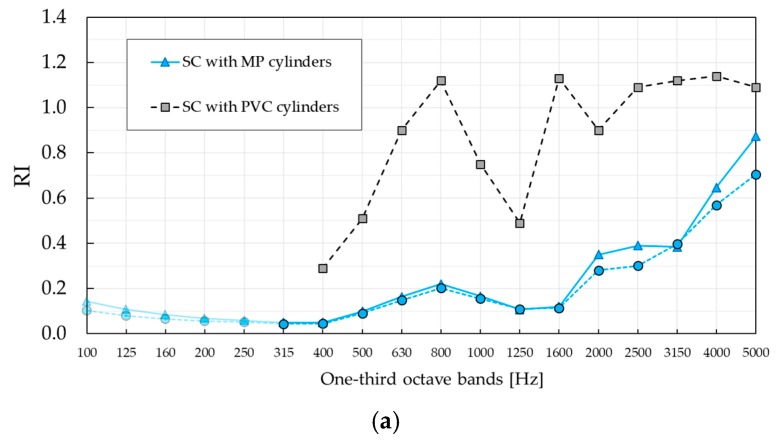

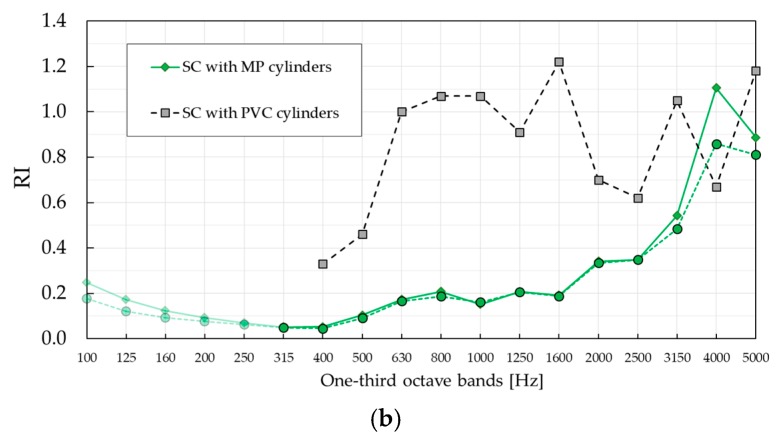
Sound reflection index reflection index (*RI)* of the SC barrier with MP cylinders measured in two different configurations. The results are compared with results of the standardised characterisation of SC barrier with PVC cylinders presented in Figure 7 in [24]. (**a**) Reflection index *RI*, configuration A; (**b**) reflection index *RI*, configuration B.

The results of standardised measurements of reflection index *RI*, computed according to Equation (2), are shown in Figure 11. As in the case of *SI* measurement, the results are also compared with those of standardized measurement of reflection index *RI* applied to the SC barrier with PVC cylinders [24]. The maximum *RI* values in Figure 11, in the Bragg gap, were well defined in both configurations of SC with PVC cylinders, with values approximately 0.9 higher than of SC with absorptive MP cylinders.

The reason is that absorptive units played a significant role in damping the effect of Bragg scattering. Compared to PVC-rigid cylinders, which provided only reflection mechanism, absorptive cylinders highlighted absorption mechanism combined with Bragg scattering, leading to a pronounced reduction of the barrier reflective properties.

**Figure 12:**
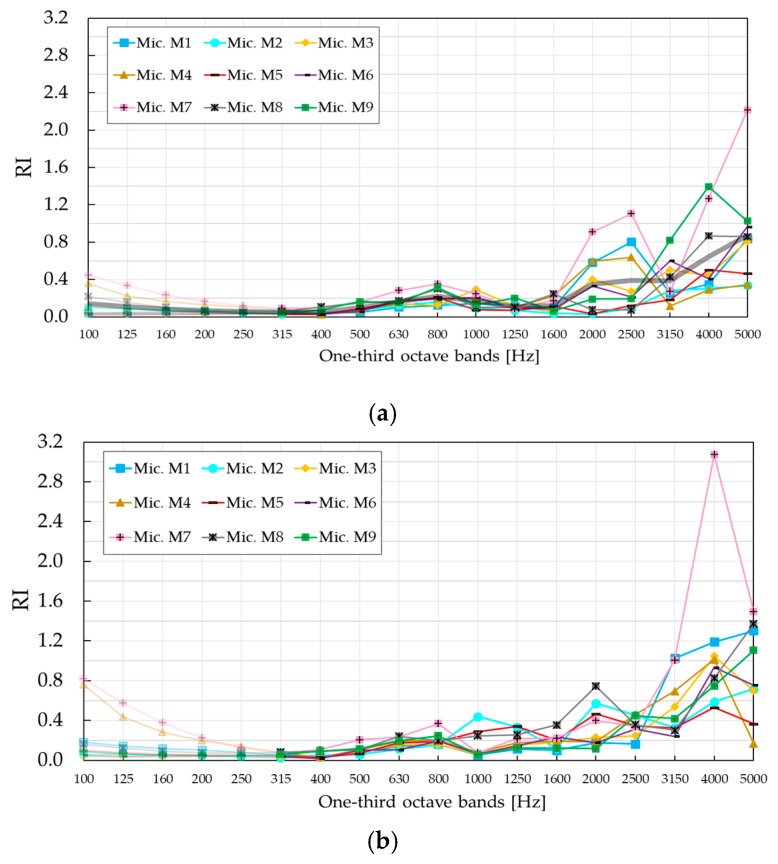
*RI* values measured at individual microphone positions M1–M9. Thick black curve shows the averaged *RI* values over all nine microphones. (**a**) Reflection index *RI*, configuration A; (**b**) reflection index *RI*, configuration B.

While SC barrier with PVC cylinders showed a remarkable difference between *RI* values evaluated in the two configurations, SC barrier with MP cylinders showed a similar trend. Namely, both configurations displayed a local maximum in the one-third octave band centred at 800 Hz and increased at higher frequencies. Small differences were visible in two frequency ranges: (i) In the one-third octave bands with the central frequency of 1250 Hz and 1600 Hz, and (ii) in the one-third octave bands, higher than the one centred at 2500 Hz. In both cases, the values were higher in configuration B. Compared to the barrier based on rigid PVC cylinders, *RI* values in the case of SC made of absorptive MP cylinders were significantly lower in nearly all one-third octave bands. The only exception was the one-third octave band centred at 4000 Hz in configuration B, where a higher *RI* was obtained for the barrier with absorptive cylinders. To investigate possible differences between different microphones, *RI* values were calculated at individual microphone positions, as shown in Figure 12. It was observed that microphone M7 exhibited an extraordinarily high value in the one-third octave band centred at 4000 Hz in configuration B, probably due to a wrong data acquisition. For that reason, results in Figure 11 were recalculated without microphone M7 and presented with blue and green curves for configurations A and B, respectively. The higher *RI* value found in SCs with MP cylinders at 4 kHz still prevailed, although after removing the effect of microphone M7, the difference was lower. The slightly higher reflectance was due to the reflectance of the barrier with PVC cylinders, which abruptly decreases at this specific third-octave band as a consequence of an interference phenomenon. An additional factor behind this behaviour might be given by the fact that the temporal window in the case of SC with PVC cylinders was centred on the arrival of the direct sound, not on the arrival of the first reflection as it is suggested by [20]. In that way, multiple scattering components coming from the surrounding cylinders were most likely windowed out. Also, two barriers are completely different, one being absorptive and the other one being completely reflective. This means that multiple scattering components did not contribute to the overall energy of reflections in the same way for two barriers. While authors in [20] highlighted the importance of the first reflection in *RI* measurements, it was the scattered energy that made a significant contribution to the overall energy of the reflections in case of the SC with MP cylinders. The reader is encouraged to observe the amplitude of the reflection components in Figure S1 in the Supporting material, where impulse responses at individual microphone positions M1–M9 in configuration A are presented.

As explained in Section 2.2.2, a single-number rating, *DL_RI_*, was introduced by EN 1793-5 standard in order to indicate the performance of the product. The calculated values were *DL_RI_* = 6.9 dB and *DL_RI_* = 6.3 dB for configuration A and B, respectively. The corresponding values in the case of SC with

PVC barrier were *DL_RI_* = 0.8 dB and *DL_RI_* = 0.5 dB for configuration A and B, respectively.

### 3.3. Three Microphone Measurements: Experimental Vs. FE Simulations

**Figure 13:**
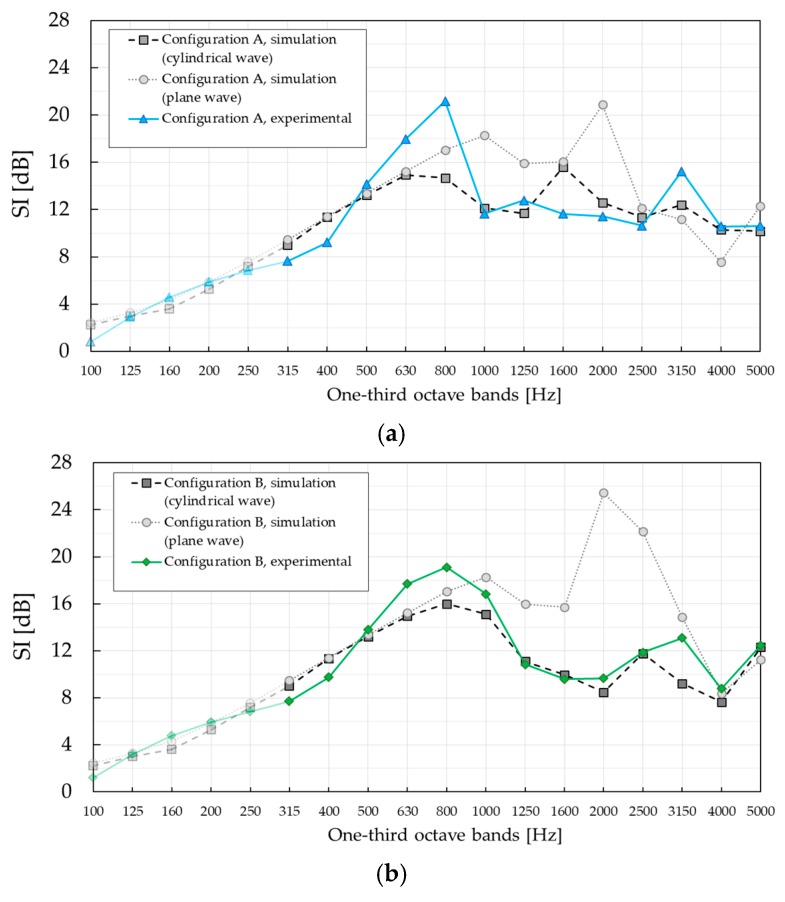
Comparison of *SI* experimental and finite element (FE) simulations results averaged over three microphone positions—M4, M5, and M6. (**a**) Sound insulation index *SI*, configuration A. (**b**) Sound insulation index *SI*, configuration B.

**Figure 14:**
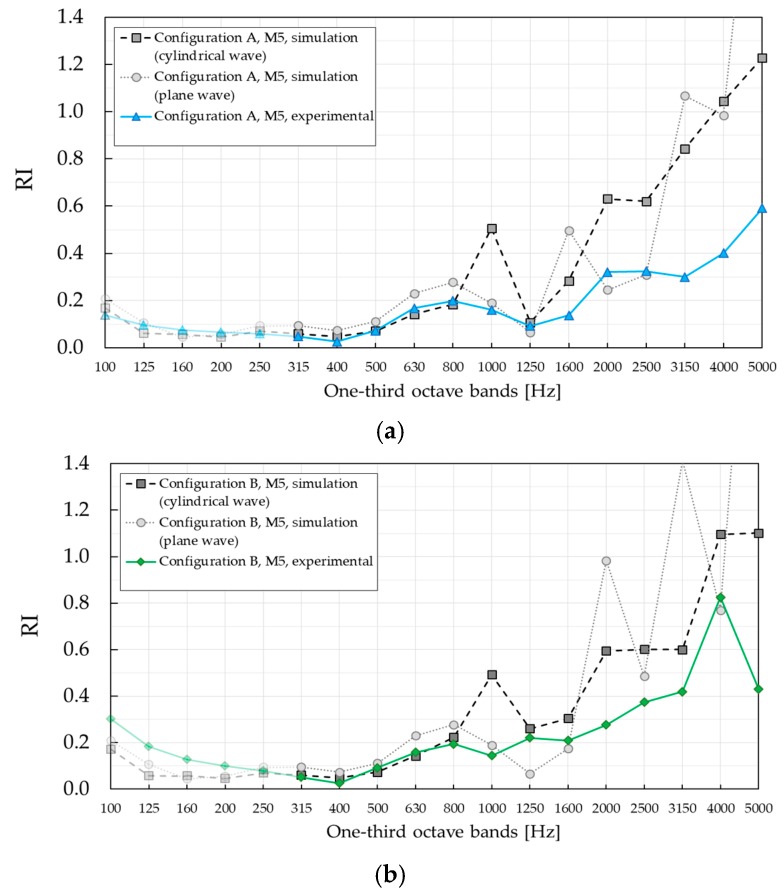
Comparison of *RI* experimental and FE simulations results averaged over three microphone positions–M4, M5 and M6. (**a**) Sound reflection index *RI*, configuration A. (**b**) Sound reflection index *RI*, configuration B.

In order to describe the experimental data, particularly the presence of the Bragg gap and diffraction effects, numerical simulations were performed in the framework of the finite element (FE) method, using *COMSOL*. Both plane and cylindrical wave-based investigations were performed using FE simulations. The former corresponded to data that one would obtain if the sample was measured in an impedance tube or at long distances from the excitation source and it was directly related to the dimensions and properties of the used materials. The latter is expected to be more realistic since the excitation source was closer to the barrier. The simulations were performed by assuming infinitely long and infinitely wide barrier for plane wave-based investigations, and infinitely long cylinders with the real width for cylindrical wave-based investigations. Results were obtained by using a mesh size small enough to guarantee both convergence and accuracy. Figure 13 and Figure 14 show a comparison of the FE simulations and experimental *SI* and *RI* results, respectively. The results were averaged over three microphone positions in the two different configurations, as shown in Figure 8. The results are presented in one–third octave bands, for consistency with the previous figures.

Regarding the results for *SI*, a better agreement between experimental data and FE simulations was obtained for the cylindrical wave condition in both configurations, except around the Bragg frequency where the simulations underestimated the measured *SI* values. For the barrier under study, based on absorptive MP cylinders, the FE simulations exhibited a quantitative agreement for most frequencies above the Bragg peak when the simulations employed cylindrical incident waves. Even though the *SI* peak values derived from the FE simulations at the Bragg frequency was lower than those derived from experimental data, the Bragg gap was still well predicted. However, the differences between the plane wave–based FE simulation results and experimental results were found to be large. In fact, the distance between the loudspeaker and the SC barrier was only 1 m, making the impinging wave at the SC barrier more cylindrical– than plane–like.

It should be pointed out that FE simulation results for *RI*, presented in Figure 14, were obtained by calculating the difference between the pressure in case with and without the SC barrier. As in case of *SI* results, *RI* values were averaged over microphones M4, M5 and M6. A good agreement between the simulated and the experimental data was observed in the frequency region up to one–third octave band with the central frequency of 800 Hz, including the Bragg gap. In higher one–third octave bands starting with one with the central frequency of 1000 Hz, a large disagreement was obtained. This issue might be related to the fact that loudspeakers at high frequencies manifest high directivity, whose radiation is significantly different from the cylindrical wave radiation. Additionally, lateral diffractions included in FE simulations with cylindrical wave–based approach were efficiently windowed out in the experimental results. As in case of *SI* measurements, plane wave–based simulations were not the appropriate approach for the given measurement setup. This is precisely why those simulations showed strong oscillations, which did not appear in the experimental results. However, both simulations and experiments show the same trend of increment of *RI* values as the frequency increased.

## 4. Conclusions

Experimental results of a standardised characterisation of a new type of sonic crystal barrier consisting of micro-perforated cylindrical shells with rubber crump, according to EN 1793-5 and EN 1793-6, were presented and discussed. Attenuation was analysed as a function of the number of layers in the barrier. The results for the barrier made of three layers were compared with reported data from a similar standardised characterisation of a sonic crystal barrier made of three layers of solid PVC cylinders. Additionally, numerical simulations were performed in the framework of multiple scattering method and the finite element method to support the measurements.

Theoretical simulations, based on finite element methods, showed a better agreement with the experimental data when a cylindrical source was employed (as opposed to a plane wave), which was attributable to the short distance between the source and the microphones in the experimental setup.

Compared to standardised characterisation of the barrier made of rigid PVC cylinders, the sonic crystal barrier consisting of absorbing micro-perforated cylindrical shells showed improved broadband sound insulation and reflection properties. Sound insulation values *SI* of 18.6 dB and 16.9 dB were obtained for two different configurations at the Bragg frequency, with single-number rating values *DL_SI_* of 12.3 dB and 12.2 dB. As opposed to the sonic crystal with rigid PVC cylinders, no pronounced constructive interference at twice the Bragg frequency was observed in the case of sonic crystal with micro-perforated shells. At individual microphone positions, *SI* values of up to 23.2 dB were obtained at Bragg frequency, as well as up to 27.6 dB in the one-third octave band centred at 1600 Hz. However, the contribution of the lower *SI* values at individual microphone positions to the averaged *SI* value in the same one-third octave band was more significant than of the higher *SI* values.

The SC barrier with micro-perforated shells exhibited significantly lower reflection values *RI* than that made of PVC cylinders, which was attributed to the energy dissipation by its absorptive building units. Reflection indices *RI* of approximately 0.2 were obtained for both configurations at the Bragg frequency, with single-number values *DL_RI_* of 6.9 dB and 6.3 dB. While reflection measurements in the case of the sonic crystal barrier with PVC cylinders were strongly affected by the position of the microphone grid, the same trend was not apparent in the barrier made of MP shells with rubber crump. Energy of multiple scattering components was found to be significant when calculating the total energy of the reflections. This was in contrary to *RI* measurements implemented in the case of the reflective sonic crystal barrier [24], where the significance of first reflections in measured impulse responses was emphasised.

The influence of leakage under the barrier and the width of the *Adrienne* temporal window was also investigated. On the one hand, leakage under the barrier had no major impact on sound insulation measurement results. Differences of 0.4 dB and 0.1 dB were obtained for single-number values with and without leakage, at the lowest three microphone positions. On the other hand, the width of the *Adrienne* temporal window would not have affected the sound insulation measurement results if ground-reflected sound transmitted through the barrier was included. When diffracted sound over the top of the barrier was included in all microphone positions, the single-number values decreased by about 2 dB in both configurations. More precisely, the diffracted sound mostly influenced the sound insulation values around the Bragg frequency.

Even though standardised EN 1793-5 and EN 1793-6 methods were not implemented on the sonic crystal barrier made with empty micro-perforated cylindrical shells in 2011, when the barrier was constructed, the absorption capability of the micro-perforated shells combined here with an inner core of rubber crumb, was preserved in both sound insulation and reflection properties. Concern that the pores of the micro-perforated shell might be clogged was unjustified.

As in the previous research by Morandi et al. [24], the *SI* measurements exhibited a strong dependency on microphone positions. The diverse sound insulation values at individual microphone positions was mainly a consequence of complex pressure patterns above the diffraction limit. Because of this strong position-dependable behaviour, but also keeping in mind the fixed distance between adjacent microphones suggested by the standardised EN 1793-5 and EN 1793-6 procedures, adjustment of their applicability to barriers based on sonic crystals requires further investigation.

## Figures and Tables

**Table 1:** Categories of airborne sound insulation according to [21].

Category	Single-Number Values [dB]
D0	Not determined
D1	<16
D2	16 to 27
D3	28 to 36
D4	>36

**Figure A1:**
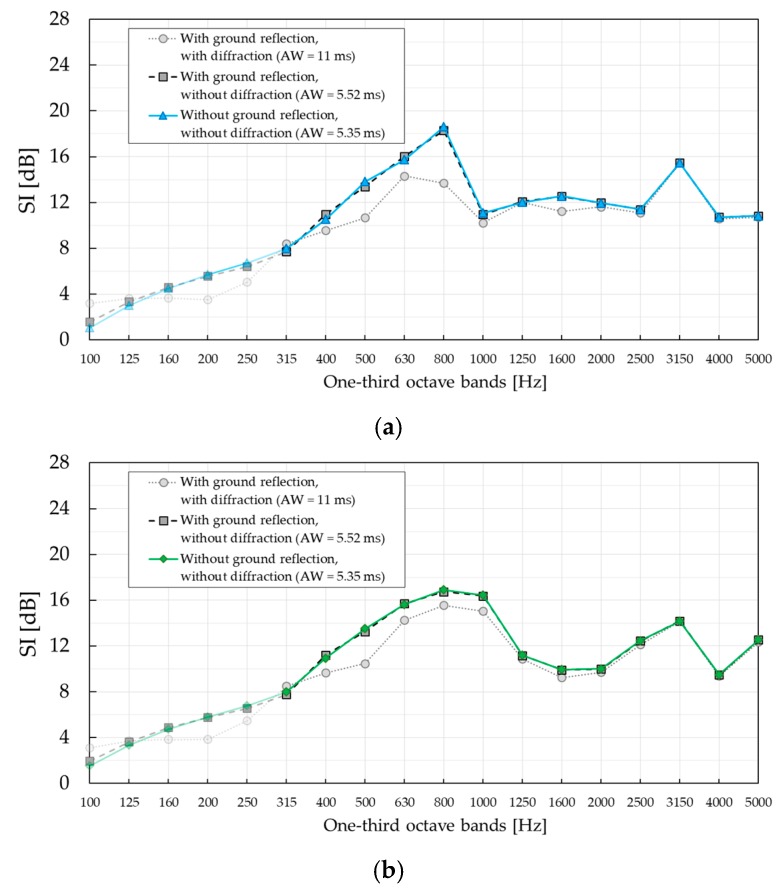
Influence of *Adrienne* temporal window width on *SI* measurements in two configurations. (**a**) Sound insulation index *SI*, configuration A; (**b**) sound insulation index *SI*, configuration B.

**Figure A2:**
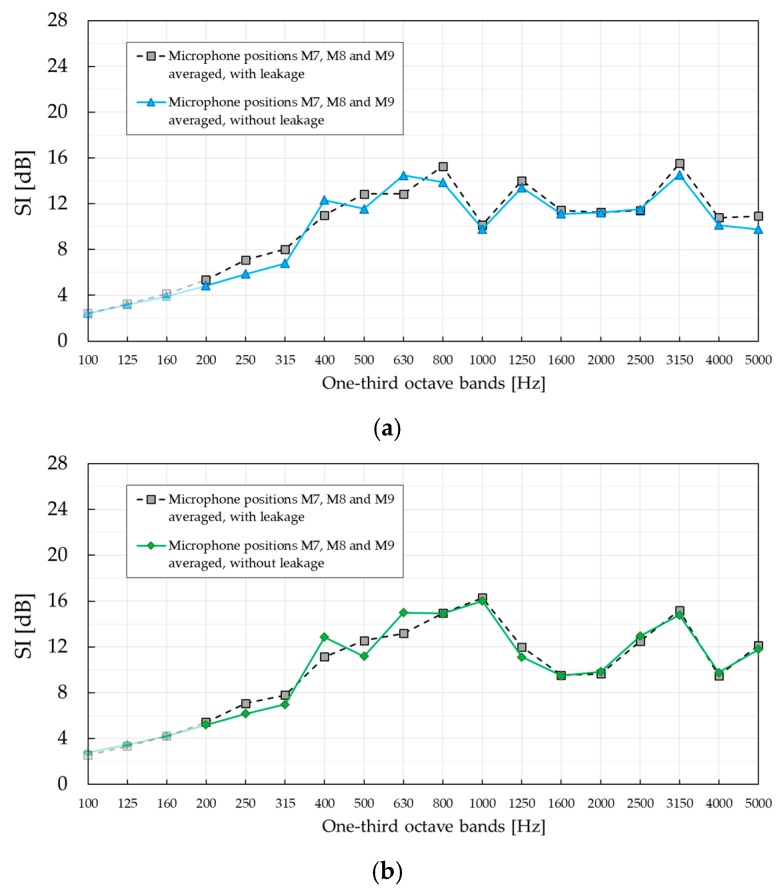
Influence of leakage under the barrier on *SI* values in the same measurement setup. The *SI* values are averages of the lowest three microphones (M7, M8, and M9) in the microphone grid. (**a**) Sound insulation index *SI*, configuration A. (**b**) Sound insulation index *SI*, configuration B.

## Supplementary Materials

The following are available online at https://www.mdpi.com/1996-1944/12/17/2806/s1, Figure S1: Impulse responses at individual microphone positions M1–M9 for standardised EN 1793-5 measurement of sound reflection index *RI* in configuration A.

## Appendix A. Modelling of the Absorbing Units

An inner crumb rubber core surrounded by an MP shell, separated by a layer of air as shown in b, was considered for the absorbing cylindrical units in all simulations. Crumb rubber was modelled as an effective fluid whose acoustic properties were as defined by the Biot–Allard model [35]. The mass density expression was derived from the simplification by Johnson et al. [36]:

(A1) $$\rho \left(\omega \right)=\rho_{0}\cdot \tau \cdot \left(1-i\cdot A\cdot \sqrt{1+\frac{\beta_{1}^{2}}{2\cdot A}}\right)$$

with

(A2) $$A=\frac{\sigma \cdot \Omega }{\omega \cdot \rho_{0}\cdot \tau }$$

where ω is the angular frequency, $\rho_{0}$ is the mass density of the filling fluid (air), and i^2^=−1. The material was defined by tortuosity τ, porosity Ω, flow resistivity σ, and empirically fitted parameter β1. The bulk modulus was calculated using the expression derived by Allard [37] from the work of Stinson [38]:

(A3) $$K\left(\omega \right)=\frac{\gamma \cdot P_{0}}{\gamma -\frac{\gamma -1}{1-i\cdot \frac{\beta_{2}^{2}}{N_{\mathrm{pr}}}\cdot A\cdot \sqrt{1+i\cdot \frac{N_{\mathrm{pr}}}{2\cdot A\cdot \beta_{2}^{2}}}}}$$

where $N_{pr}=0.706$ is the Prandtl number and γ=1.4 is the specific heat ratio of the filling fluid. An additional parameter $\beta_{2}$ was included in the characterisation of the porous material. The values of the material properties were the same as in [9], which reports the characterisation of sonic crystals with the same type of crumb rubber: τ=1.54, Ω=0.541, σ = 3318.6 rayls/m, $\beta_{1}$ = 1.58, and $\beta_{2}$ = 0.7.

The outer MP cylindrical shell was configured through an interior impedance condition, where the acoustic impedance of the flat MP plate was introduced as a parameter. The sheets employed in the barrier consisted of aluminium plates with a thickness of t = 1 mm and holes that can be approximated as slits with a half-length of b = 553.3 μm and half-width of a = 47.5 mm [9]. The expression of its acoustic impedance [9,26,39,40] was:

(A4) $$Z_{\mathrm{MP}}=\frac{i\cdot \omega \cdot \rho_{0}\cdot t}{\sigma_{M}}\cdot {\left(1-\frac{\tanh\left(s\cdot \sqrt{i}\right)}{s\cdot \sqrt{i}}\right)}^{-1}+\frac{2}{\sigma_{M}}\cdot \sqrt{2\cdot \eta_{0}\cdot \omega \cdot \rho_{0}}+\frac{i\cdot \omega \cdot \rho_{0}}{\sigma_{M}}\cdot 0.96\cdot \sqrt{a\cdot b}\cdot \left(1-1.25\cdot \sqrt{\sigma_{M}}\right)$$

where $\eta_{0}$ is the dynamic viscosity of air, $\sigma_{M}$ is the perforation ratio ($\sigma_{M}=3.43\%$ for the employed sheets), and s is the perforation constant defined as:

(A5) $$s=a\cdot \sqrt{\frac{\omega \cdot \rho_{0}}{\eta_{0}}}.$$

Rigid cylinders, used here for comparison purposes, were modelled by removing their interior domain from the simulation and setting a rigid boundary condition.

## Appendix B. Influence of Width of the *Addrienne* Temporal Window

At any individual microphone position, it was the direct sound that arrived first when an excitation signal was emitted from the loudspeaker. After the direct sound, ground-reflected sound transmitted through the barrier and a diffracted sound over the top of the barrier is usually detected, in an order that depends on the microphone position. In the top row of microphones in the microphone grid (M1, M2, and M3), it was the diffracted sound that arrived before the ground-reflected sound. In the lowest row of microphones (M7, M8, and M9), the situation was the opposite. In the middle (M4, M5, and M6), it was the ground-reflected sound that arrived only 0.3 ms before the diffracted sound. To investigate the possible influence of the width of the *Adrienne* temporal window (AW) on *SI* values, the results of a standardised characterisation with AW = 5.35 ms are compared with two additional cases—AW = 5.52 ms and AW = 11 ms. The results are presented in Figure A1.

In the case where a width of 5.52 ms was used for the *Adrienne* temporal window, ground reflections transmitted through the SC barrier were included at all microphone positions except those in the topmost row of the microphone grid (M1, M2, and M3). This was as expected, regardless of the case, since at those microphone positions the diffracted sound always arrives before ground-reflected sound. To account for the diffracted sound, the *Adrienne* temporal window width was set to 11 ms. In that way, diffracted sound was captured at all microphone positions, even those in the lowest row of the microphone grid.

The influence of the ground reflection transmitted through the SC barrier was negligible. On the other hand, diffracted sound affected the *SI* values, especially in the frequency range around the Bragg gap. The single-number values derived in configuration A when AW = 5.35 ms, AW = 5.52 ms, and AW = 11 ms for the *Adrienne* temporal window were used were 12.3 dB, 12.2 dB, and 10.2 dB, respectively. In configuration B, the single-number values were 12.2 dB, 12.2 dB, and 10.4 dB. It is also important to be aware of different low-frequency limits when the width of the temporal window varies. When 5.35 ms and 5.52 ms were used, the low-frequency limit was the one-third octave band centred at 315 Hz, whereas in the case of 11 ms, the low-frequency limit was one-third octave band centred at 160 Hz. In general, a lower frequency limit results in lower *SI* single numbers. If the same frequency range was used in the case of 11 ms as in the case of 5.35 ms and 5.52 ms, the single-number value would have been 11.3 dB.

## Appendix C. Influence of Leakage Under the Barrier

The influence of leakage under the barrier was investigated by sealing the space between the flat metal base and the ground with bricks, see a. Measurement was then repeated after the bricks were removed. A comparison of the *SI* values in the two cases is shown in Figure A2. The *SI* values are averages of the lowest three microphones (M7, M8, and M9) in the microphone grid. At these microphone positions, the ground-reflected sound components transmitted through and those passing under the barrier will arrive before the diffracted sound over the top of the barrier. By setting a proper width of the *Adrienne* temporal window, all diffracted sound components will be excluded from the calculation of *SI* values. The presence of the ground-reflected sound components transmitted through the barrier should not be of great concern since they are present in both measurements, with and without leakage. A modified *Adrienne* window with a width of 7.84 ms was used for this purpose, whose low-frequency limit was f_MIN_ = 164 Hz. Accordingly, the results are presented starting from the one-third octave band with a central frequency of 200 Hz.

As shown in Figure A2, the influence of leakage under the barrier in either configuration was insignificant. In a way, this was expected, since the barrier thickness resulted in a large path that attenuated the leakage component and introduced insignificant interference when the leakage component met with the direct sound. In this way, the thickness of the SC barrier introduced enough attenuation to make the influence of the sound passing under it negligible. Single-number values of 10.7 dB and 11.2 dB in configuration A were obtained without and with the leakage, respectively. Similarly, the single-number values in configuration B were 11.1 dB and 11.3 dB. When there is leakage, higher or lower values could be a consequence of the constructive or destructive interference between the direct sound and the sound passing under the barrier.
