# Supplementary material for: Sound Insulation and Reflection Properties of Sonic Crystal Barrier Based on Micro-Perforated Cylinders
Source: Materials (Basel). 2019 Aug 31;12(17):2806. doi: 10.3390/ma12172806 (PMC6747850; doi:10.3390/ma12172806)
Supplement: Supplementary file 1 [file materials-12-02806-s001.pdf]

materials

MDPI

Supplementary Materials

Sound Insulation and Reflection Properties of Sonic Crystal Barrier Based on Micro-Perforated Cylinders

Stefan M. Dimitrijević [1,2], Víctor M. García-Chocano [3], Francisco Cervera [3], Emelie Roth [1] and José Sánchez-Dehesa [3,*]

[1] Structor Akustik AB, Solnavägen 4, 113 65 Stockholm, Sweden
[2] School of Electrical Engineering, University of Belgrade, Bulevar K. Aleksandra 73, 11000 Belgrade, Serbia
[3] Department of Electronic Engineering, Universitat Politècnica de València, Camino de Vera s/n, ES-46022 Valencia, Spain
* Correspondence: jsdehesa@upv.es; Tel.: +34-63-0353-603

Received: 30 June 2019; Accepted: 27 August 2019; Published: date

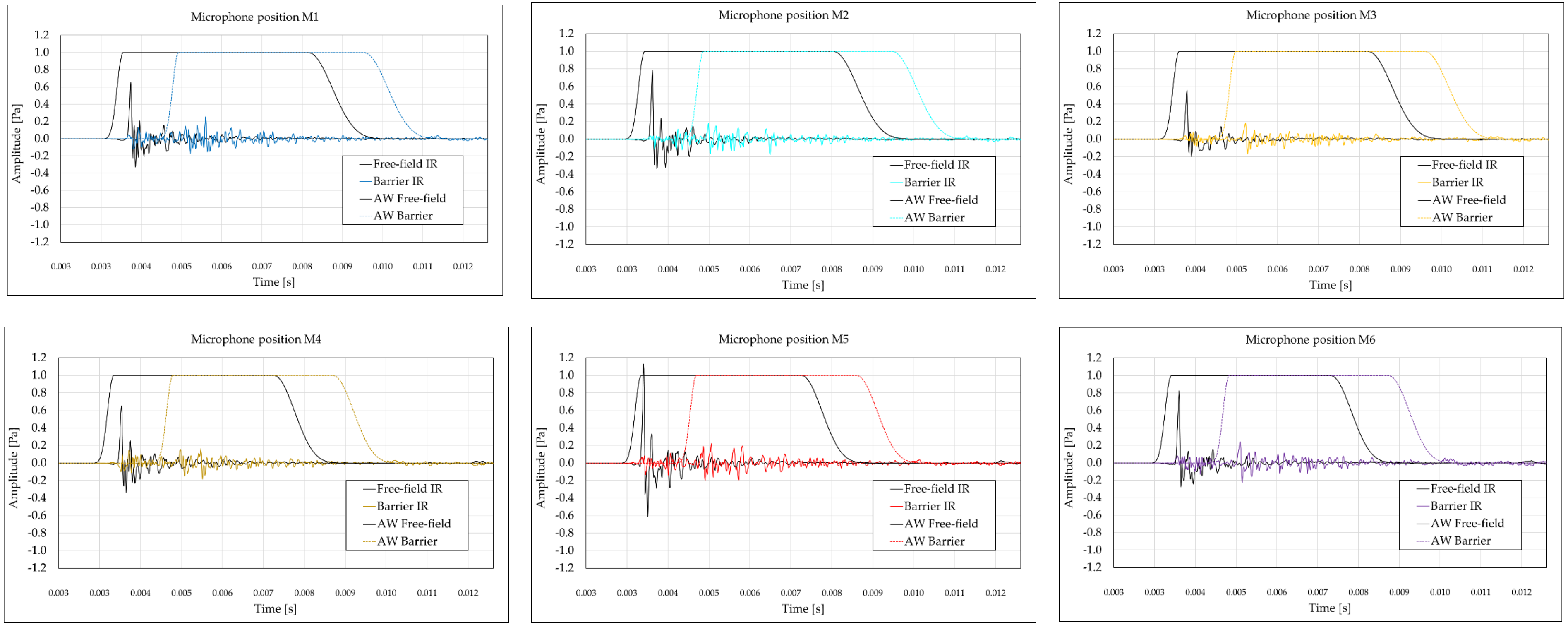
Microphone position M1
Microphone position M2
Microphone position M3
Microphone position M4
Microphone position M5
Microphone position M6
Amplitude [Pa]
Time [s]
1.2
1.0
0.8
0.6
0.4
0.2
0.0
-0.2
-0.4
-0.6
-0.8
-1.0
-1.2
0.003
0.003
0.004
0.005
0.006
0.007
0.008
0.009
0.010
0.011
0.012
Free-field IR
Barrier IR
AW Free-field
AW Barrier

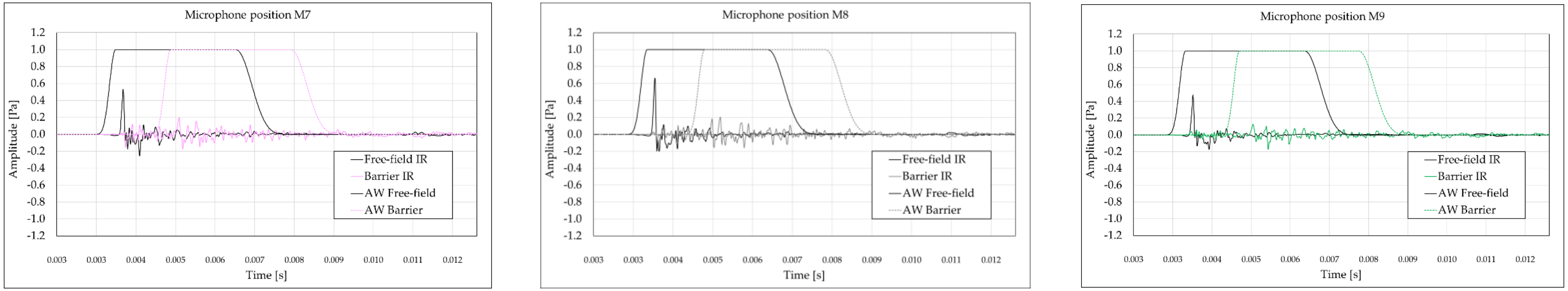

Figure S1. Impulse responses at individual microphone positions M1 – M9 for standardised EN 1793–5 measurement of sound reflection index *RI* in configuration A.

materials

© 2019 by the authors. Submitted for possible open access publication under the terms and conditions of the Creative Commons Attribution (CC BY) license (http://creativecommons.org/licenses/by/4.0/).
